# Sub-Standard Pharmaceutical Services in Private Healthcare Facilities Serving Low-Income Settlements in Nairobi County, Kenya

**DOI:** 10.3390/pharmacy7040167

**Published:** 2019-12-05

**Authors:** Kennedy Abuga, Dennis Ongarora, Jamlick Karumbi, Millicent Olulo, Warnyta Minnaard, Isaac Kibwage

**Affiliations:** 1 Department of Pharmaceutical Chemistry, University of Nairobi, P.O. Box 19676, Nairobi 00202, Kenya; dennis.bagwasi@uonbi.ac.ke (D.O.); ikibwage@gmail.com (I.K.); 2 Division of Health Products and Technologies, Ministry of Health, P.O. Box 30016, Nairobi 00100, Kenya; karumbij@gmail.com; 3 PharmAccess Foundation, P.O. Box 6711, Nairobi 00100, Kenya; m.olulo@pharmaccess.or.ke; 4 Stichting PharmAccess International, AHTC, Tower 4C, Paasheuvelweg 25, 1105 BP Amsterdam, The Netherlands; w.minnaard@pharmaccess.org; 5 Administration, Planning and Development, Egerton University, Njoro, P.O. Box 536, Egerton 20115, Kenya

**Keywords:** pharmaceutical service, brand preference, drug order, competition, drug disposal

## Abstract

**Background:** Quality pharmaceutical services are an integral part of primary healthcare and a key determinant of patient outcomes. The study focuses on pharmaceutical service delivery among private healthcare facilities serving informal settlements within Nairobi County, Kenya and aims at understanding the drug procurement practices, task-shifting and ethical issues associated with drug brand preference, competition and disposal of expired drugs. **Methods:** Forty-five private facilities comprising of hospitals, nursing homes, health centres, medical centres, clinics and pharmacies were recruited through purposive sampling. Structured electronic questionnaires were administered to 45 respondents working within the study facilities over an 8-week period. **Results:** About 50% of personnel carrying out drug procurement belonged to non-pharmaceutical cadres namely; doctors, clinical officers, nurses and pharmacy assistants. Drug brand preferences among healthcare facilities and patients were mainly pegged on perceived quality and price. Unethical business competition practices were recorded, including poor professional demeanour and waiver of consultation fees veiled to undercut colleagues. Government subsidized drugs were sold at 100% profit in fifty percent of the facilities stocking them. In 44% of the facilities, the disposal of expired drugs was not in conformity to existing government regulatory guidelines. **Conclusions:** There is extensive task-shifting and delegation of pharmaceutical services to non-pharmaceutical cadres and poor observance of ethical guidelines in private facilities. Strict enforcement of regulations is required for optimal practices.

## 1. Introduction

In Kenya, healthcare services are provided by public and private healthcare facilities at different hierarchical levels. It is a legal requirement for suppliers to sell drugs strictly to licensed healthcare practitioners and registered premises. Further, depending on the license granted, a facility is required to employ defined cadres of staff [1,2,3,4]. Facilities licensed to offer pharmaceutical services are required to employ a pharmacist or pharmaceutical technologist, being the two cadres licensed by the local regulator, the Pharmacy and Poisons Board (PPB) to practice pharmacy in Kenya. Pharmaceutical technologists possess an intermediate level diploma qualification while pharmacists undergo degree training. Current regulations, however, do not require that all pharmaceutical services have to be provided by these cadres. First, facilities solely licensed to offer health services do not face this requirement but are nevertheless permitted to keep a limited range of drugs for treatment purposes. Second, facilities with a pharmacy license are not explicitly forbidden to employ other staff to ‘support’ the pharmacist or pharmaceutical technologist. As a consequence, they commonly employ untrained personnel who gradually attain sufficient experience to perform limited functions otherwise reserved for the legitimate professionals [5,6]. These personnel members constitute the unofficial cadre of ‘pharmacy assistants’ who routinely work alongside the licensed actors in an act of task-shifting and delegation.

Such task-shifting is subject to growing debate. On the one hand, task-shifting is seen as a key strategy to address the substantial shortage of trained health workers in low-income settings [7,8] while on the other hand, this practice may affect the quality of services provided. Research on the extent to which the latter applies to pharmaceutical services is scant. A study on 270 public health facilities in Tanzania suggests that such task-shifting ‘may have negative implications both for availability of medicines and quality of clinical care’, but acknowledges that it ‘does not yield any conclusion on possible causality’ [9]. Other studies have demonstrated that knowledge of service providers in low-income settlements is often inadequate, and so is appropriateness of treatments, diagnostic accuracy, prescription processing and competitive behaviour [10]. Much is, however, yet unknown about the impact of task-shifting on service quality, particularly so for private sector providers. Enhancing understanding of this matter is highly needed to guide policy framework and context for stimulating task-shifting.

The current study was designed to fill this gap by focusing on private drug-selling facilities in informal settlements (low-income areas) in Nairobi. Informal settlements account for about 60% of the population in Nairobi County. Their inhabitants are characterised as part of the bottom of the (wealth) pyramid (BOP) ecosystem [11,12,13]. They prefer private to public facilities due to proximity, friendly interactions, shorter queue times, and flexibility of pricing and payment [14].

The purpose of the study was to describe private sector pharmaceutical services with respect to licensure of facilities, drug procurement behaviour and various indicators of service quality. A better understanding of pharmaceutical services delivery provides an insight into the quality of care and possible areas of interventions to improve patient outcomes.

## 2. Materials and Methods

### 2.1. Study Design, Area, Sites and Sampling

This descriptive cross-sectional survey was nested in a larger descriptive study conducted in Nairobi County, Kenya between September and December 2016 as previously reported in a related paper by Ongarora et al. using similar tools within the study setting described [15].

### 2.2. Recruitment and Informed Consent of Participants

During recruitment, the owners of the healthcare facilities were approached and informed that study interviews were targeted at personnel responsible for drug procurement at the facility. Potential participants were fully informed about the purpose of the study, possible risks and benefits at the time of enrolment. They were however, permitted to make a conscious decision to decline participation or withdraw from the study at any time. Willing healthcare facilities thus provided written informed consent and signed a contract under the terms of research for the duration of the study. The identity of participating facilities was blinded by assigning unique identifiers in the database and reports.

### 2.3. Pharmaceutical Services

The pharmaceutical services targeted during the survey were derived from the statutory guidelines, policy documents and literature reports on related studies [5,16]. Drug supply chain management aspects such as licensing, executing cadres, opinions on supplier services and brand preferences were evaluated. Additionally, the prevailing practices with regard to handling of government subsidized items (such as antimalarials and selected family planning commodities), dispensing of drugs, business competition, stocking and use of rapid diagnostic test kits at the facilities, and disposal of expired drugs were investigated. The specific questions incorporated in the questionnaire are listed in Table 1.

### 2.4. Data Collection and Management

Trained field agents used structured, electronic questionnaires designed to capture pertinent information on the prevailing practice and status of the pharmaceutical services provided by the study facilities. Data collected include: cadre of the respondent, license that was used to order drugs and the person who actually made the drug order. For these variables total responses (denominator) were 45 as only one option is applicable per facility. Other indicators were modes of ordering drugs, and reasons for stocking a given brand and changing a drug supplier; perceived reasons that made patients buy a given brand; stocking and use of rapid diagnostics test (RDT) kits; and modes of disposal of expired drugs. For the latter, it was possible to obtain multiple responses for the same variable, thus yielding a denominator greater than 45. 

The respondents were interviewed on four occasions at weeks 1, 3, 6 and 8, whereof data were captured at each time point. All data were stored in data files with password secured access to ensure security and confidentiality.

### 2.5. Data Analysis

Data analysis was carried out using Microsoft Excel spreadsheets. Descriptive analysis was undertaken for pharmaceutical services indicators. All the indicators were summarized by their frequency (presented using frequency tables) of occurrence and also in percentages. No attempt of analysing associations was done.

### 2.6. Ethical Approval

The University of Nairobi-Kenyatta National Hospital, Ethics and Research Committee (UoN-KNH ERC) granted approval for the study (approval number KNH-ERC/A/371), as per legal requirements in Kenya.

## 3. Results

### 3.1. Characteristics of Facilities

A total of 45 facilities participated in the study. They consisted of seven hospitals, two nursing and maternity homes, two health centres, four medical centres, 18 clinics and 12 pharmacies. The operational definitions of these facility types are listed in applicable laws of Kenya [1,2]. Only two clinics and one hospital possessed pharmacy licenses (premises and practice) from the Pharmacy and Poisons Board (PPB) which authorized them to perform retail pharmaceutical services. In conformity with the Kenyan law, the standalone pharmacies also operated under these two licenses.

The facility names displayed on the business premises were compared to their licenses, whereby it was observed that 20/45 facilities (44%) had licensed designations unmatched with the type and level of operations. Clinics and medical centres had names that depicted a higher level of care, while those of the nursing homes and six hospitals implied a lower level classification. The standalone pharmacies were named using the suffixes: pharmacy, chemist(s), pharmaceuticals, pharmcare and pharma.

The distribution of the professional cadres of the respondents is illustrated in Table 2. The pharmaceutical cadres (pharmacists and pharmaceutical technologists) were only encountered in community pharmacies.

### 3.2. Drugs Procurement Behaviour

The respondents were subjected to questions about the prevailing practices associated with drug orders as described hereafter.

#### 3.2.1. Licenses Used for Drug Orders

Pharmaceutical technologists represented the highest number of participating licensures in drug ordering (13) followed by doctors (10). Pharmacists’ licenses were used to place orders in eight facilities while one hospital used the facility license (unspecified) (Table 2).

#### 3.2.2. Cadres Placing Drugs Orders

Pharmaceutical technologists placed orders in 13/45 (29%) facilities while pharmacists and doctors participated evenly in this task. Nurses performed this task in 18% of the facilities, whilst a few facilities engaged clinical officers (4), ‘pharmacy assistants’ (1) and administrators (1) as shown in Table 2. 

#### 3.2.3. Methods of Making Drugs Orders

Majority (56%) of drugs orders were made by facilities reaching out to their suppliers via telephone when in need of stock replenishment. The second most popular method also involved telephony, as suppliers prompted facilities to make orders (Table 2). The use of information technology (IT) in effecting drugs orders through e-mail or other means accounted for 16 (5%) of the responses.

#### 3.2.4. Quality Perceptions of Supplier Services

Over 99% of the respondents perceived their current supplier as offering good quality services. This was attributed to proper supplier licensure (56%) or quality products as inferred from lack of complaints from patients (43%). However, when looking for a new supplier, price was the main consideration (27%), while perceived quality and drugs assortment contributed 21% and 12% respectively to such choice. Other factors mentioned were the availability of credit facilities, speed of delivery of drugs orders, accessibility and relationship in decreasing order.

#### 3.2.5. Brand Preference

Perceived product quality and pricing emerged as the main determinants of brand preference for a given drug (Table 2). The respondents further provided information on their choice to procure original branded drugs or generic products. The five major influencing factors, as measured by the frequency of responses obtained, were prescription specifications (20%), price (16%), patient needs (12%), drugs resistance (11%) and efficacy (10%).

Similarly, the healthcare facilities were probed on the attributes by which patients purchased drugs at the facility. The results show that drug prices and perceived quality influenced patients’ brand preferences most (Table 2).

### 3.3. In Situ Practices

The themes presented in this section were designed to discern in-house practices that impact on the quality of pharmaceutical services and ethical practices.

#### 3.3.1. Sale of Government Subsidised Drugs

Twelve facilities (27%) stocked government subsidised drugs, mostly antimalarials, whereas only two facilities had contraceptive pills. They mentioned four main reasons why patients were interested in buying subsidised drugs from their facilities namely: proximity of the private health facility vs. public facilities (3), patients’ perceived (better) quality of drugs supplied at private facilities (3), lack of drugs at public facilities (3) or long waiting times at public facilities (2). Six facilities levied 100% profit margins on these products while the other six applied a 30–40% return.

#### 3.3.2. Influence of Competition on Practice

A large majority (39/45, 87%) of the respondents considered neighbouring facilities as their main competitors, with one facility mentioning herbal clinics. Competitor facilities were perceived to edge on variety, quality of services and cheaper pricing of products. Facilities with in-built medical laboratories and those accredited by funded medical schemes such as National Hospital Insurance Fund (NHIF) were thought to possess a competitive advantage. Four respondents cited unethical behaviours such as close proximity of premises (without due regard to the catchment capacity), strained professional interactions with colleagues and waiver of consultation fees. There was one claim of the existence of improperly licensed pharmacies which further engaged in unspecified malpractices.

#### 3.3.3. Prescription Substitution

When processing prescriptions, the majority of the respondents preferred to order missing drugs from their regular supplier or buy them from a nearby facility, after seeking concurrence of the client. Four respondents (9%) said that they could substitute the prescribed original branded drug for a generic or even perform therapeutic substitution.

#### 3.3.4. Prescription and Dispensing Practices

The respondents were asked about the basis for dispensing drugs to their patients. Forty-four respondents gave two alternatives whereby 26/45 (58%) indicated that they dispensed drugs either on presentation of a prescription or performing diagnostic tests before prescribing and dispensing. A total of 18/45 (40%) facilities, half of which were standalone pharmacies, indicated that they dispensed on the basis of a prescription or patient demand (without prescription).

#### 3.3.5. Use of Rapid Diagnostic Test Kits 

The rapid diagnostic test (RDT) kits encountered in the facilities were those for malaria, pregnancy, HIV, VDRL (syphilis) and *Helicobacter pylori*. Twenty facilities stocked RDT kits while 39 performed the actual tests (Table 2) implying that some facilities asked clients to procure them elsewhere. Forty facilities indicated using the RDT as the basis for prescription.

The respondents were asked what they would do if a patient with a negative test still demanded for drugs. All except two facilities said they would counsel the patient and request for further tests or recommend a referral to another health facility. One respondent however said they would dispense anti-malarials upon demand but not for other drugs.

#### 3.3.6. Disposal of Expired Drugs

In Kenya, the Pharmacy and Poisons Board (PPB) has prescribed guidelines for the disposal of expired drugs [16]. This obliges incineration of the expired items by the concerned players under the supervision of the PPB or contracting licensed companies for this purpose. The fate of expired drugs in the facilities under study is summarized in Table 2.

The disaggregated responses for the ‘other methods’ category revealed that 22 submitted the expired items to a government facility for disposal while 11 contracted a licensed firm for the service. The remaining 13 either stored them in a separate area within the facility or requested the PPB to carry out the disposal.

## 4. Discussion

The pharmaceutical services recorded during the study indicate a deviation from recommended practice for the aspects considered. Several reports on the practices of retail pharmacies have shown propensity to non-adherence to regulations [7]. This is the first study combining all the types of facilities with respect to pharmaceutical practices.

Comparison of premises names and the functional licenses of the facilities showed 20 mismatches, which might be misleading to clients. Correlation between the names and the services offered is important because clients are likely to choose a facility by virtue of its name, size and reputation rather than licensing [17]. Furthermore, use of misleading words is prohibited under existing laws [2].

The number of pharmaceutical personnel acting as respondents (11) could not be rationalised with that against whose licenses were used to order drugs (21) or who participated in the ordering process (22). This discrepancy indicates that some facilities may be ‘renting’ licenses from the pharmacy professionals to facilitate operations [18].

Task-shifting and delegation was observed with half of the healthcare facilities placing drugs orders through non-pharmaceutical cadres. This may be an expedient way of controlling costs for business survival [7,19]. This is not surprising given the lack of role clarity and blurred professional boundaries as previously reported [18]. It is disturbing that ‘pharmacy assistants’, a cadre that is not recognized by the laws of Kenya, were offering pharmaceutical services in some facilities in consonance with previous studies [11,18].

As is typical for Kenyan facilities, drugs orders were placed through telephone calls to or from the suppliers [20]. However, e-mail and other automated or digitized methods were not commonly used within the study population contrary to developed countries, where integrated software is used to manage inventory and prescriptions with improved efficiency and accountability [21,22]. Furthermore, the slum study setting may not readily embrace ICT platforms for operations due to cost and capacity building constraints.

Studies have demonstrated that stocking and prescribing patterns are significantly determined by supplier aggressiveness rather than logical professional judgment [18]. Brand preference based on quality (Table 2) was most likely perceptual as corroborated by studies conducted in Pakistan and Saudi Arabia [23,24], since the facilities have no suitable mechanisms of determining the product quality. For patients, brand preference is influenced by price since they mostly pay out-of-pocket for healthcare [15].

Prescription of specific brands by physicians has been recognized as a major factor in determining the products dispensed at retail outlets. Individual prescribers may prefer specific innovator brands due to previous experience, brand loyalty and perceptual poor performance by generics. In some cases, direct financial inducements by drug companies are employed to influence prescribers to promote branded drug products which undermines generic prescribing and increases costs of treatment to patients. Counter incentives by the health plan financiers to promote generic prescribing have also been used in the United States with similar ethical dilemmas [25]. The study brought out different factors such as prescriber and patient needs, price and efficacy. The stocking behaviour of pharmacies is likely to be modelled on prescriber actions since they are obliged to satisfy prescription needs of their clients [26,27]. Patients on the other hand tend to consider the cost of the treatment, perceived quality and efficacy. This outcome is in tandem with the observations of Guttier et al. [28] that linked patient preference of generic products to perceived safety, efficacy, previous experience and financial ability. Some patients however, may prefer branded products due to previous experience or influence from the attending physician [29].

Prescription substitution as encountered during the study is most likely a shielding mechanism to keep customers from competitors. This practice has been a point of conflict between prescribers, dispensing personnel and the patient which highlights the ethical issues surrounding the practice [25,30,31,32].

The artemisinin-combination-therapy (ACT) antimalarials and contraceptive pills are supplied on government subsidy for improved availability and affordability [33,34]. It is disturbing that half of the facilities that stocked these drugs were making 100% profits for these items, thus impeding affordability.

Stiff competition from local players inspired by the desire for client retention and business survival was a major factor influencing ethics of practice. This underscores the need for the regulators to rationalize catchment areas using established models to curb unhealthy competition, promote professional colleagueship and ethical adherence [35].

The practice environment in resource limited settings, compels the facilities to use rapid diagnostic test (RDT) kits since they present a relatively inexpensive and convenient method to carry out diagnosis and generate appropriate prescriptions. The use of these devices as a basis for prescription in the study facilities is a right step towards rational practices. Indeed, RDT kits are actively promoted for malaria, HIV and pregnancy diagnosis [36].

With regard to the disposal of expired drugs, it is worrying that 44% of facilities used unorthodox methods such as throwing them away. Drugs disposed through general garbage may cause environmental pollution. Furthermore, they eventually reach the dumpsites where they are liable to misuse by street families. Twelve facilities passed on near-expiry drugs to other facilities for use on unsuspecting clients. Some of the responses provided about drugs disposal were doubtful under the prevailing practice environment in Kenya. For instance, private facilities cannot submit expired drugs to a government facility or the PPB for disposal as claimed. It appears, the drug disposal mechanisms prescribed by the PPB are ineffective as the majority of the facilities did not employ them and therefore may require review [16].

## 5. Study Limitations

The study was carried out as part of a larger survey on the study sites thus limiting its coverage of some pharmaceutical practice aspects, especially patient- and drug-specific services. The study suffers other limitations. First, there was no attempt to check any correlation or association as the data collected was not sufficient. The study design was not powered to test associations since the aim was to describe the practice rather than infer association or correlation. Second, since patients were not interviewed during the study, the data on patients’ brand preference may not be reliable since it reflects respondent (health care provider) perceptions, this might have biased our findings but nevertheless, offers an insight on how the providers of medicines to the BOP segment perceive their clients. Finally, the findings of the present study may not be transferable to rural BOP settings where the practice dynamics are likely to be different.

## 6. Conclusions

The prevailing practice of pharmaceutical services offered by private health facilities serving BOP patients were characterized by task shifting and delegation, unhealthy competition and unorthodox drug disposal practices. Even though the Pharmacy and Poisons Board (PPB) stipulates proper licensing and supervision of operations by pharmacy qualified staff (pharmacists and pharmaceutical technologists) in all pharmacies, the on-site practices seem far from ideal. The private sector is laden with problems of manpower shortages, blurred boundaries on scope of practice and poor interprofessional coordination. Thus, remedial regulatory interventions need to be instituted to curb discordance between policy and practice.

## Figures and Tables

**Table 1:** Questionnaire items used for data collection.

Who is responsible for ordering drugs at your facility? Under which license are the drugs procured in your facility? How does the order process typically work at your facility? How do you mainly decide what brand to procure for a particular drug molecule? What are the attributes by which your patients purchase their drugs from you? How do you determine to buy original branded vs branded generics vs generic drugs? What is your main motivation to procure from your current supplier(s)? Do you think your current supplier(s) provide quality and how have you determined this? What would you look for in a potential new drugs supplier? What is your cadre as per your license? What name is written on the business premises? Is your license for a clinic, pharmacy or hospital? Do you sell drugs that are available for semi-free (subsidised) in the public sector? If yes, what is the reason? What are the profit margins (%)? Which public sector drugs do you sell most? Who do you see as your main competitor when selling drugs to your patients? Why is it considered a competitor? What do you do if a patient requires a drug that you do not have in stock? What advice do patients require when buying drugs? Do you sell any diagnostic rapid tests to be used by the client? Which ones? Do you perform any of those tests yourself? Do you prescribe drugs based on such a test? What do you do when a test result is negative and a patient keeps asking for drugs?

**Table 2:** Pharmaceutical services indicators.

Service Indicator	Parameter Values						Total
**Cadres/License**	**Pharmacist (%)**	**Pharm. Tech. (%)**	**Doctor (%)**	**Clinical Officer (%)**	**Nurse (%)**	**Other cadres (%)**	
Respondent (person)	2 (4)	9 (20)	3 (7)	8 (18)	20 (44)	3 (7)	45 (100)
License used to order drugs	8 (18)	13 (29)	10 (22)	4 (9)	9 (20)	1 (2)	45 (100)
Person ordering drugs	9 (20)	13 (29)	9 (20)	4 (9)	8 (18)	2 (4)	45 (100)
Methods of Ordering *	**HCP Phone**	**Supplier Phone**	**HCP Visit to Supplier**	**SREP Visit to Facility**	**E-Mail**	**IT-Automated**	
	165 (56)	81 (27)	33 (11)	2 (1)	12 (4)	4 (1)	297 (100)
Choice of New Supplier *	**Price**	**Quality**	**Variety**	**Reliability of Supply**	**Speed of Delivery**	**Others**	
	166 (27)	130 (21)	76 (12)	70 (11)	669(11)	108 (18)	616 (100)
**Brand Preference**	**Price**	**Brand**	**Quality**	**Efficacy**	**Availability**	**Patient Preference**	
Facility *	129 (27)	38 (8)	139 (29)	80 (16)	45 (9)	52 (11)	483 (100)
Patient *	142 (30)	40 (9)	110 (24)	69 (15)	66 (14)	39 (8)	466 (100)
Rapid Diagnostic Test Kits *	**Stock RDT Kits**	**Perform Diagnostic Test**	**Use Results as Basis for Prescription**				
	20	39	40				N/A
Disposal of Expired Drugs *	**Return to Supplier**	**Throw Away**	**Pass on to other Facilities**	**No Expiries Occurred**	**Other Methods**		
	57 (25)	42 (18)	12 (5)	72 (32)	46 (20)		229 (100)

HCP = healthcare provider, IT = information technology, N/A = not applicable, Pharm. Tech. = pharmaceutical technologist, RDT = rapid diagnostic test, SREP = sales representative. * The total is more than 45 because cumulative responses have been used. These responses were obtained from the respondents during each of the four interview occasions.
